# NVIDIA’s “Chat with RTX” Custom Large Language Model and Personalized AI Chatbot Augments the Value of Electronic Dermatology Reference Material

**DOI:** 10.2196/58396

**Published:** 2024-07-24

**Authors:** Maged N Kamel Boulos, Robert Dellavalle

**Affiliations:** 1 School of Medicine University of Lisbon Lisbon Portugal; 2 Department of Dermatology University of Minnesota Medical School Minneapolis, MN United States

**Keywords:** AI chatbots, artificial intelligence, AI, generative AI, large language models, dermatology, education, self-study, NVIDIA RTX, retrieval-augmented generation, RAG

## Abstract

This paper demonstrates a new, promising method using generative artificial intelligence (AI) to augment the educational value of electronic textbooks and research papers (locally stored on user’s machine) and maximize their potential for self-study, in a way that goes beyond the standard electronic search and indexing that is already available in all of these textbooks and files. The presented method runs fully locally on the user’s machine, is generally affordable, and does not require high technical expertise to set up and customize with the user’s own content.

## Introduction

Artificial intelligence (AI) chatbots powered by large language models (LLMs) can potentially improve clinical learning experiences and promote self-paced study—for example, by summarizing large amounts of text data, such as a collection of research articles—thus helping users to instantly identify key information in large bodies of literature [1]. However, uploading copyrighted and other sensitive content for processing by web-based (externally hosted) chatbots may prove to be problematic; for example, it may violate applicable license agreements and regulations.

On the other hand, running fully locally hosted and managed instances of LLMs and their associated end-user interfaces (eg, ChatGPT [OpenAI]) requires very large investments (starting at tens of thousands of US dollars) in hardware and infrastructure [2]. This cost has decreased with the launch of NVIDIA’s free “Chat with RTX” tech demo download in February 2024 [3], which can be used to build custom LLMs and personalized AI chatbots. “Chat with RTX” runs fully locally on relatively inexpensive laptops and does not require high technical expertise to set up and customize with the user’s own content.

This paper describes a novel use of “Chat with RTX” to build a cloud-independent, dermatology self-study AI chatbot that can work with, and enhance the educational value of, electronic textbooks and research papers locally stored on the user’s computer without uploading them to any remote server. Electronic textbooks and research papers are often acquired in .pdf format. The presented AI chatbot offers additional functionality beyond that of the standard electronic search and indexing that is already available in .pdf files, such as the abilities (1) to link, synthesize, and summarize at a single location related information scattered across different book chapters and multiple papers and (2) to generate knowledge-testing quizzes with answers.

## Building a Dermatology Self-Study AI Chatbot

NVIDIA’s “Chat with RTX” (version 0.2; installer file size: 35 GB, downloaded on March 7, 2024, from the NVIDIA website [4]) was installed on an ASUS TUF Dash F15 (2022) laptop running Microsoft Windows 11 (version 23H2) on an Intel i7-12650H CPU and NVIDIA RTX 3070 Laptop GPU (driver version: 537.42), with 8 GB of GDDR6 VRAM, 32 GB of DDR5 system RAM, and 2 TB of SSD storage. A video tutorial demonstrating a typical installation procedure is available at on the web [5].

“Chat with RTX” comes bundled with the Mistral 7B LLM [6] and allows users to customize the chatbot by importing their own datasets (.txt, .pdf, and .doc files) or YouTube video links (in this case, it will fetch and use the corresponding transcripts from YouTube). It is a retrieval-augmented generation (RAG) application, whereby the user’s datasets become an external knowledgebase to an existing LLM [7]. The system runs locally on the user’s machine (as a local server), and imported user files never leave the user’s machine, which is a very important feature (and in some cases, a legal requirement) when working with private, confidential (eg, clinical notes), and copyrighted material. (While it is possible to upload files for similar processing by some web-based chatbots for free or for a small subscription fee, this can often violate copyright conditions or patient privacy by having material sent to, processed by, and possibly stored on third-party servers.)

**Figure 1:**
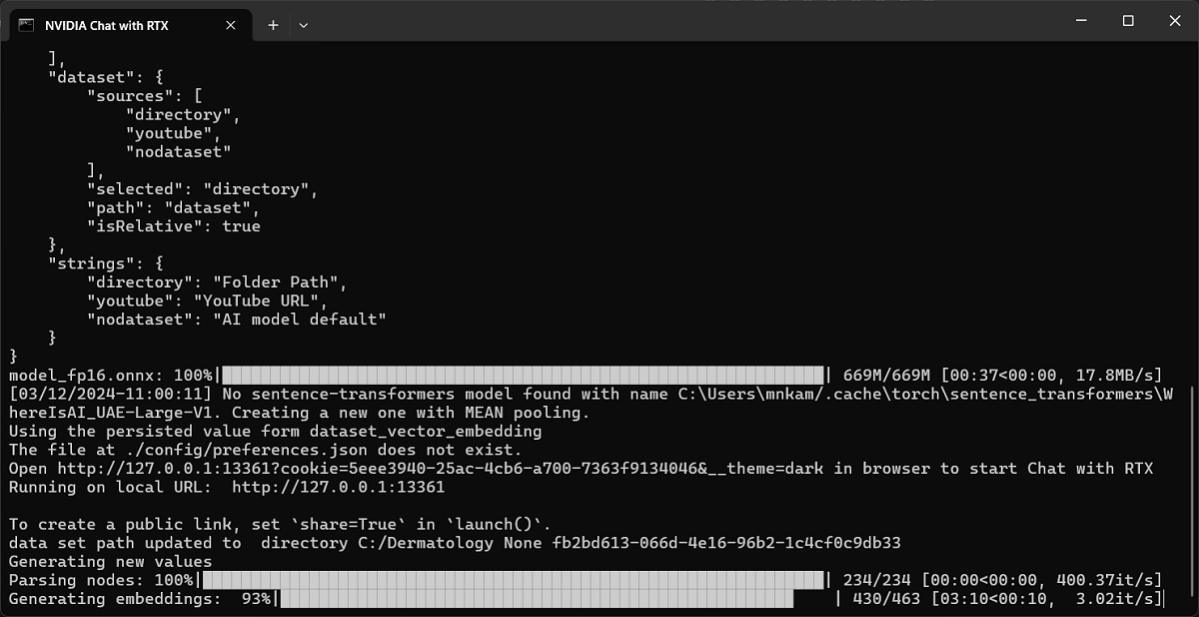
Importing a locally stored dermatology textbook file into NVIDIA's "Chat with RTX".

A local electronic copy of a dermatology textbook for testing purposes (an 11 MB .pdf file with 234 pages) was imported [8]. “Chat with RTX” took about 3 minutes (on the particular laptop configuration used in this demonstration) to parse the textbook and generate embeddings (mathematical representations of words in a high-dimensional space; Figure 1). It is possible to import more than 1 document or textbook by putting all the documents to be imported into 1 folder and pointing “Chat with RTX” to this folder. This can provide better topical coverage and results. However, it is not advisable to import too many documents, as the software can take hours, days, or even months to complete processing them. A faster RTX GPU with more VRAM (eg, 12 GB or 24 GB) can significantly help speed up this processing task. It should be noted that parsing only needs to be done once when the custom AI chatbot is first created and that the generated embeddings are saved to the local SSD for subsequent uses or until the chatbot’s content is changed.

**Figure 2:**
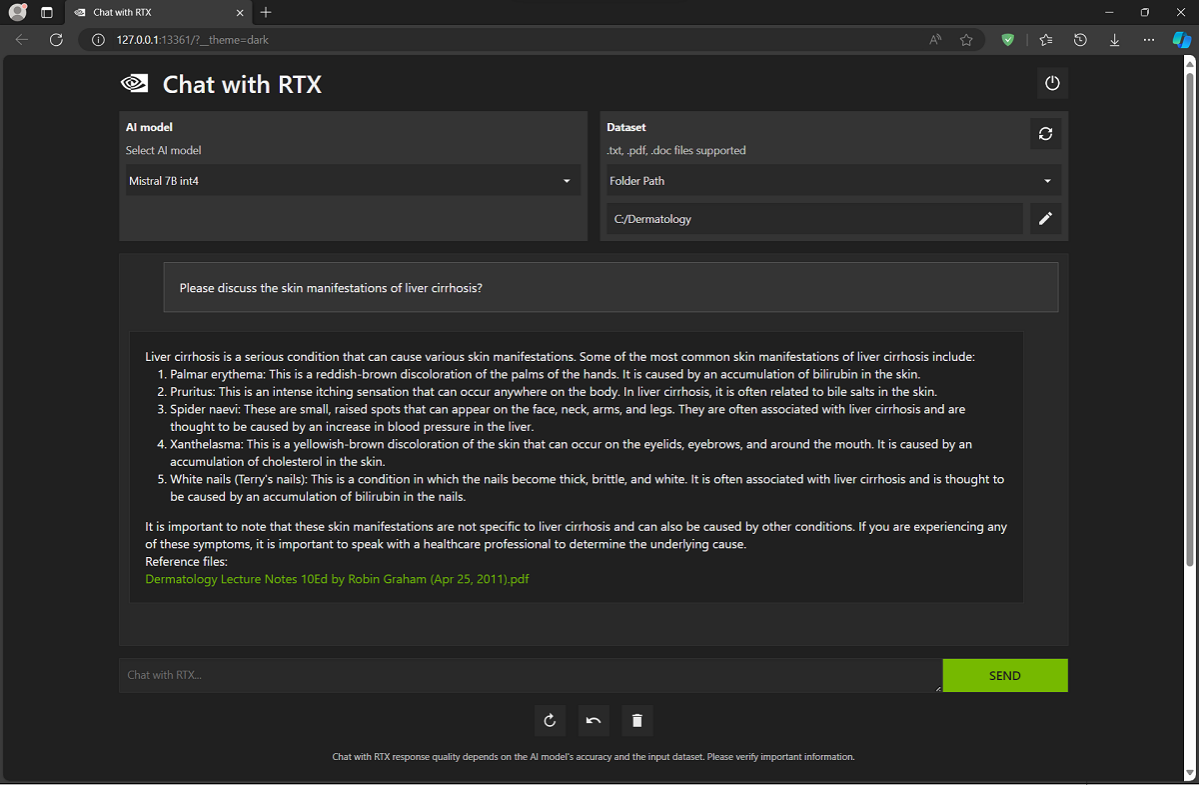
NVIDIA's "Chat with RTX" response to prompt to discuss the skin manifestations of liver cirrhosis. Note the cited source listed at the bottom of the chatbot answer.

**Figure 3:**
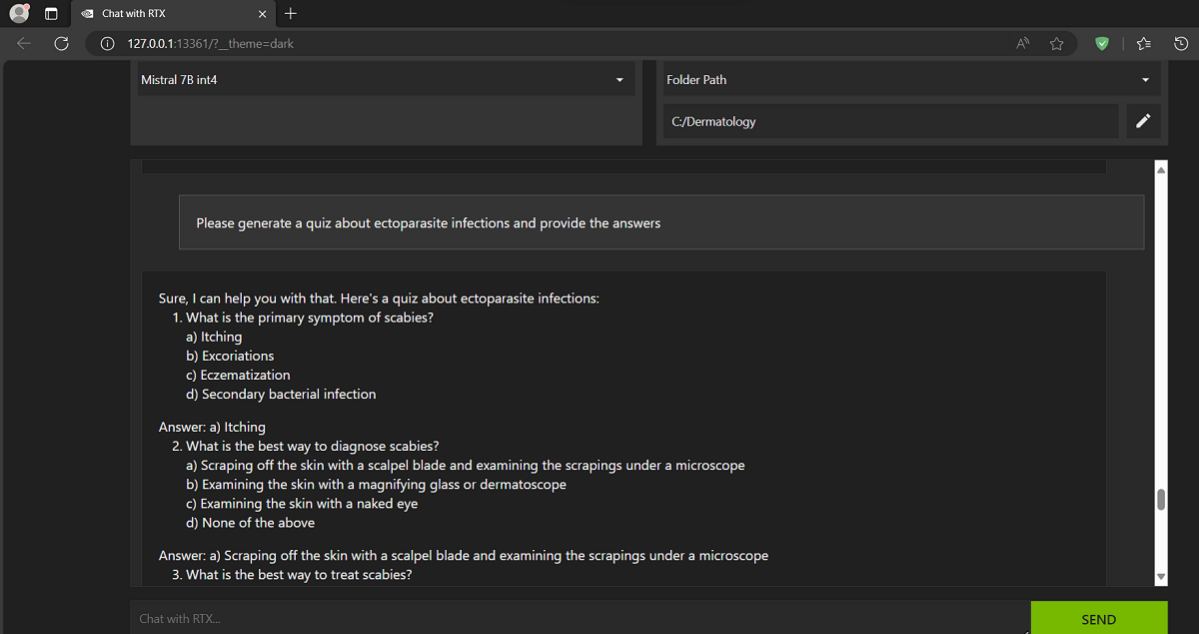
NVIDIA's "Chat with RTX" (customized with a local copy of a dermatology textbook) was prompted to generate a quiz (with answers) about ectoparasite infections.

The newly created custom dermatology AI chatbot was prompted to discuss the skin manifestations of liver cirrhosis (Figure 2) and to generate a quiz (with answers) about ectoparasite infections (Figure 3), among other queries. “Chat with RTX” provided reasonably good answers and would end each answer by citing the “Reference files” it used in generating the answer. The latter is an important feature when the user’s dataset contains more than 1 document, for example, multiple textbooks or papers, with each answer being attributed to its correct source(s). In comparison, the Aeyeconsult web-based chatbot by Singer et al [9] answers eye care–related questions using only verified ophthalmology textbooks as data and always cites its sources.

## Current Limitations

“Chat with RTX” is still an early tech demo with rough edges and limitations, such as (at the time of writing) its currently less than ideal user interface for importing and parsing user content and its relatively costly consumer hardware requirements, which may put it out of reach for some users [10,11]. It is also prone to hallucinations (albeit to a lesser degree than non-RAG systems) and other inconsistencies of generative AI [12,13]. However, as is the case with other digital technologies, this emerging consumer technology (software and hardware) will continue to improve and become increasingly more affordable over time.

## Discussion and Future Directions

The goal of this exercise was not to compare “Chat with RTX” answers with those of a human dermatology expert, but rather to demonstrate a new, promising way to increase the educational value of electronic textbooks and research papers (locally stored on the user’s machine) and maximize their potential for self-study, in a way that goes beyond the standard electronic search and indexing that is already available in all of these textbooks and .pdf files. “Chat with RTX” does this by serving as an intelligent personal clinical tutor, for example, by summarizing important facts, linking and synthesizing related bits across different book chapters and papers, developing study themes spanning multiple chapters, and generating quizzes (and answers for marking them) for the user to test their own knowledge and understanding of a subject, as briefly demonstrated in this paper (Figures 2 and 3).

General purpose LLMs such as OpenAI’s GPT-4o are not optimized for clinical use and are prone to generating hallucinatory information. RAG as used in “Chat with RTX” enables the creation of custom LLMs and personalized AI chatbots that are specifically and comprehensively trained using handpicked corpora of quality, evidence-based medical texts that sufficiently cover a given clinical area of specialism [12,13].

Cloud independence is another notable feature of AI chatbot implementation using “Chat with RTX.” The ability to run fully offline not only protects copyrighted and other sensitive data but also offers more flexibility to users, by allowing them to run the software in places and situations where there is no internet connection.

Although promising, a dermatology self-study AI chatbot such as the one presented in this paper will need to undergo formal testing, evaluation, and refining or fine-tuning as necessary before it can be signed off for mainstream use. Testing and evaluation should cover critical aspects of these chatbots such as accuracy and impact on student learning outcomes [1], among others, and should be revisited whenever the underlying software implementation or medical content are updated.

In the future, publishers might consider bundling electronic dermatology (and other clinical specialty) textbooks with custom self-study AI chatbots to offer a superior service to their readers.

## Abbreviations

AI: artificial intelligence
LLM: large language model
RAG: retrieval-augmented generation
